# Polycyclic aromatic hydrocarbons: determinants of urinary 1-hydroxypyrene glucuronide concentration and risk of colorectal cancer in the Shanghai Women’s Health Study

**DOI:** 10.1186/1471-2407-13-282

**Published:** 2013-06-11

**Authors:** Jonathan N Hofmann, Linda M Liao, Paul T Strickland, Xiao-Ou Shu, Gong Yang, Bu-Tian Ji, Hong-Lan Li, Nathaniel Rothman, Farin Kamangar, Yu-Tang Gao, Wei Zheng, Wong-Ho Chow

**Affiliations:** 1 Occupational and Environmental Epidemiology Branch, Division of Cancer Epidemiology and Genetics, National Cancer Institute, 9609 Medical Center Drive, Room 6E132, MSC 9771, Bethesda, MD 20892, USA; 2 Bloomberg School of Public Health, Johns Hopkins University, Baltimore, MD, USA; 3 Department of Medicine, Vanderbilt University, School of Medicine, Nashville, TN, USA; 4 Department of Epidemiology, Shanghai Cancer Institute, Shanghai, China; 5 Department of Public Health Analysis, School of Community Health and Policy, Morgan State University, Baltimore, MD, USA; 6 Department of Epidemiology, The University of Texas MD Anderson Cancer Center, Houston, TX, USA

**Keywords:** 1-hydroxypyrene glucuronide, Polycyclic aromatic hydrocarbons, Colorectal cancer, China

## Abstract

**Background:**

Associations between polycyclic aromatic hydrocarbons (PAHs) and colorectal cancer have been reported previously but few studies have characterized PAH exposure using biological measurements. We evaluated colorectal cancer risk in relation to urinary concentration of 1-hydroxypyrene glucuronide (1-OHPG), a polycyclic aromatic hydrocarbon (PAH) metabolite, and assessed determinants of PAH exposure among controls in the Shanghai Women’s Health Study (SWHS).

**Methods:**

Concentrations of 1-OHPG were measured in spot urine samples collected from 343 colorectal cancer cases and 343 individually matched controls. Questionnaires were administered to collect information on demographic characteristics and reported exposures. Odds ratios were calculated for risk of colorectal cancer in relation to quartiles of urinary 1-OHPG concentration. Potential determinants of natural log-transformed urinary 1-OHPG concentration were evaluated among a combined sample of controls from this study and another nested case–control study in the SWHS (N_total_=652).

**Results:**

No statistically significant differences in risk of colorectal cancer by urinary 1-OHPG levels were observed. Among controls, the median (interquartile range) urinary 1-OHPG concentration was 2.01 pmol/mL (0.95-4.09). Active and passive smoking, using coal as a cooking fuel, eating foods that were cooked well done, and recent consumption of fried dough (e.g., yóutiáo) were associated with elevated levels of 1-OHPG, though only active smoking and fried dough consumption achieved statistical significance in multivariate analyses.

**Conclusions:**

This study does not provide evidence of an association between urinary levels of 1-OHPG and risk of colorectal cancer among women. Several environmental and dietary sources of PAH exposure were identified. Overall, the levels of 1-OHPG in this population of predominantly non-smoking women were considerably higher than levels typically observed among non-smokers in Europe, North America, and other developed regions.

## Background

Polycyclic aromatic hydrocarbons (PAHs) are byproducts of incomplete combustion of organic materials. Individuals may be exposed to PAHs through various environmental sources including tobacco smoke, ambient air pollution, and consumption of grilled foods [1]. High levels of PAH exposure have been observed for various occupational activities including coal gasification, coke production, aluminum smelting, coal-tar distillation, and paving and roofing with coal-tar pitch [2]. The International Agency for Research on Cancer has classified PAH exposure in these occupational settings as a Group 1 carcinogen (“carcinogenic to humans”).

Colorectal cancer is the third most common type of incident cancer in the world among both men and women [3]. There is some evidence of an increased risk of colorectal cancer in relation to PAH exposure. Colorectal cancer mortality was elevated among PAH-exposed gas furnace workers in an occupational cohort study in Germany [4], and several case–control studies have reported associations between estimated dietary intake of benzo(a)pyrene, a particular PAH compound, and risk of colorectal adenoma [5-8]. In a prospective investigation within the Prostate, Lung, Colorectal and Ovarian Cancer Screening Trial, higher estimated dietary benzo(a)pyrene intake was associated with an increased risk of incident rectal adenoma but not colon adenoma [9]. Furthermore, active cigarette smoking, a major source of PAH exposure, has also been consistently associated with an increased risk of colorectal cancer [10].

However, few studies have evaluated the relationship between PAH exposure biomarkers and risk of colorectal adenoma [11] or colorectal cancer [12]. In this nested case–control study, we evaluated the risk of colorectal cancer in relation to urinary concentration of 1-hydroxypyrene glucuronide (1-OHPG), a metabolite of pyrene and an established biomarker of PAH exposure [13], among participants in the Shanghai Women’s Health Study. As a secondary analysis, we evaluated the relations between urinary 1-OHPG concentration and potential determinants of PAH exposure among a combined sample of controls from this study and a related nested case–control study of gastric cancer.

## Methods

### Study participants

Colorectal cancer cases and matched controls were selected from among participants in the Shanghai Women’s Health Study (SWHS), a prospective cohort of approximately 75,000 women enrolled between 1997 and 2000 [14]. Of the 65,574 women in the SWHS cohort who provided spot urine samples at enrollment, 347 were diagnosed with incident colorectal cancer through December 2005. One control was individually matched to each case based on age (±2 years), date of sample collection (within 1 month), menopausal status at sample collection, time of sample collection (morning or afternoon), and time interval since last meal (within two hours). Four of the colorectal cancer cases were subsequently determined to have been misdiagnosed; these cases and the corresponding matched controls were excluded from the analyses of colorectal cancer risk, leaving 343 cases (colon cancers, N=205; rectal cancers, N=138) and matched controls.

A total of 652 controls from the SWHS cohort were included in the analyses of determinants of urinary 1-OHPG concentration (347 controls from the colorectal cancer study, and 305 controls from a nested case–control study of gastric cancer for which samples were analyzed contemporaneously at the same laboratory).

### Data and urine specimen collection

The design and collection of data and specimens in the SWHS has been described [14]. Briefly, women between 40–70 years of age were recruited from selected urban communities in Shanghai, China from 1997 to 2000. All subjects provided written informed consent, and the study protocols were approved by the Institutional Review Boards of the National Cancer Institute, Vanderbilt University, and the Shanghai Cancer Institute. After obtaining informed consent, subjects completed a self-administered questionnaire and an in-person interview. Information was collected on demographic characteristics, active and passive cigarette smoking, diet and cooking practices, occupational history, and various other factors. Height and weight were measured at the time of the in-person interview.

Subjects were also asked to provide a spot urine sample, which was kept cold and processed within 6 hours for long-term storage. Another questionnaire was completed at the time of sample collection to obtain additional information about diet, smoking, and medication use within the previous week and the 24-hour period prior to urine collection.

### Outcome ascertainment

Colorectal cancer cases were identified through linkage with the Shanghai Cancer Registry and the Shanghai Vital Statistics Unit records, and in biennial follow-up visits with participants. Information on the date of cancer diagnosis was collected, and diagnoses were verified by reviewing medical charts and diagnostic slides. Incident cases of colon and rectal cancers diagnosed through December 2005 were included in this analysis.

### Measurement of urinary 1-OHPG concentration

Measurements of urinary concentration of 1-OHPG were performed using immunoaffinity chromatography and synchronous fluorescence spectroscopy [13]. Assays were performed in batches of 20 including laboratory QC samples and blinded duplicate samples in each batch. The coefficient of variation (CV) for replicate measurements of urinary 1-OHPG concentration across batches using aliquots from a single quality control pool was 10.6%. In an analysis of blinded duplicate samples from 30 subjects, the Spearman rank correlation coefficient for paired 1-OHPG measurements was 0.82.

The assay limit of detection was 0.1 pmol/mL. For measurements below the limit of detection (7.6% and 7.3% of cases and controls, respectively), a value of 0.05 pmol/mL was assigned.

### Statistical analysis

Conditional logistic regression analyses were performed to estimate odds ratios (ORs) and 95% confidence intervals (CIs) for risk of colorectal cancer in relation to creatinine-adjusted urinary 1-OHPG concentration, which was categorized as quartiles based on the distribution in controls. Statistical tests for trend were performed by modeling the within-category medians as a continuous parameter. We also evaluated colorectal cancer risk in relation to creatinine-adjusted urinary 1-OHPG concentration as a continuous variable; as in previous studies [15], a one unit change was defined as half the difference between the 25^th^ and 75^th^ percentile measurements in controls. Analyses were performed with and without adjustment for the following covariates: age (years); education level; smoking status; aspirin use; estimated fruit, vegetable, and folate intake; measured body mass index (BMI); and leisure time and occupational physical activity [in metabolic equivalent hours per week (MET-h/wk), as estimated in ref. [16]. Because intra-individual variability in 1-hydroxypyrene levels was modestly reduced in first morning void urine samples compared to 24-hour urine samples after adjustment for creatinine [17], we used creatinine-adjusted 1-OHPG concentrations in our main analyses. However, to evaluate whether correcting 1-OHPG measurements for creatinine concentration had any effect on the resulting risk estimates, we performed sensitivity analyses using 1-OHPG concentrations without correction for creatinine, and with creatinine concentration included as an independent variable in the statistical model, as recommended by Barr et al. [18]. Several analyses were performed to assess whether the association differed by time from sample collection to case diagnosis, including an analysis restricted to cases (and corresponding matched controls) diagnosed two or more years after sample collection, and analyses stratified by duration of follow-up (below/above the median time of 3.8 years from sample collection to diagnosis, and above the 75^th^ percentile of follow-up time to diagnosis of 5.6 years). We also conducted analyses stratified by tumor location (colon vs. rectal) and menopausal status.

As secondary analyses, we evaluated urinary 1-OHPG concentration as a dependent variable in relation to selected exposures that were suspected a priori to contribute to PAH exposure. These analyses were conducted among a combined set of control subjects (N=652). Measurements of 1-OHPG concentration were corrected for creatinine concentration, and data were natural log-transformed to achieve a normal distribution. Results are reported as the geometric mean (GM) and 95% CI within each exposure category. Self-reported exposures from the baseline questionnaire that were evaluated included active and passive smoking status, cooking fuel type, cooking ventilation conditions, and food preparation methods. We also evaluated smoking and food preparation methods in the last week and last 24 hours prior to sample collection (as reported on the sample collection questionnaire). Independent t-tests were used to evaluate each variable separately in relation to 1-OHPG concentration. We also performed multivariate analyses for exposures reported on the baseline questionnaire (Model 1) and exposures reported for the last 24 hours on the sample collection questionnaire (Model 2); both models were adjusted for age in years, level of education, measured BMI, and study sample (i.e., controls from the colorectal cancer study or gastric cancer study).

All statistical analyses were performed using Stata version 10 (StataCorp LP, College Station, TX). Findings were considered statistically significant if two-sided *P*-values were < 0.05.

## Results

### Colorectal cancer

Colorectal cancer cases and matched controls were similar in level of education, marital status, fruit and vegetable consumption, BMI, folate intake, and physical activity (Table 1). Smoking was uncommon in general among study participants, but was slightly less common among cases than among controls (2% and 5%, respectively). Urinary levels of 1-OHPG, with or without adjustment for creatinine, were not significantly different between cases and controls. The geometric means (95% CIs) of creatinine-adjusted 1-OHPG concentration in urine were 0.15 (95% CI 0.13-0.17) and 0.16 (95% CI 0.14-0.18) μmol/mol creatinine for cases and matched controls, respectively (*P* = 0.4, paired *t*-test).

**Table 1:** **Frequencies of selected characteristics for colorectal cancer cases and matched controls**^**a**^

**Characteristic**	**Cases (N=343)**	**Controls (N=343)**
Age in years, mean (SD)	59.0 (8.4)	59.1 (8.4)
Level of education		
Elementary school or less	144 (42.0)	154 (44.9)
Middle school	99 (28.9)	87 (25.4)
High school	68 (19.8)	60 (17.5)
College	32 (9.3)	42 (12.2)
Marital status		
Not married	61 (17.8)	62 (18.1)
Married	282 (82.2)	281 (81.9)
Menopausal status		
Pre-menopausal	74 (21.6)	77 (22.5)
Post-menopausal	268 (78.4)	266 (77.6)
Ever smoked cigarettes (≥1/day for 6+ months)		
No	336 (98.0)	326 (95.0)
Yes	7 (2.0)	17 (5.0)
Aspirin use in last year (3+/wk for >2 mo)		
No	334 (97.4)	329 (95.9)
Yes	9 (2.6)	14 (4.1)
Vegetable intake, g/week, mean (SD)	303 (179)	284 (160)
Fruit intake, g/week, mean (SD)	243 (167)	248 (177)
Folate intake, μg/day, mean (SD)	288 (100)	290 (99)
Measured body mass index (BMI), mean (SD)	24.7 (3.2)	24.8 (3.4)
Leisure time physical activity (MET hrs/wk), mean (SD)	110 (44)	108 (46)
Cumulative occupational energy expenditure (kJ/min), mean (SD)	243 (99)	230 (98)

Abbreviations: *SD* standard deviation, *BMI* body mass index, *MET hrs/wk* metabolic equivalent hours per week.

^a^ Reported as frequencies (%) within each category unless otherwise specified.

No statistically significant differences in risk of colorectal cancer by creatinine-adjusted 1-OHPG concentration were observed; adjustment for selected covariates did not have any appreciable effect on risk estimates (Table 2). Results were similar when husband’s smoking status was included in the multivariate analysis, and when smoking variables were not included in the statistical model (data not shown). Risk of colon cancer, but not rectal cancer, appeared to decrease slightly with increasing levels of creatinine-adjusted 1-OHPG; however, this trend was not statistically significant when 1-OHPG was analyzed as a continuous variable. Risk estimates did not change appreciably after exclusion of cases (and corresponding matched controls) diagnosed within two years of sample collection, and we did not observe differences in risk by menopausal status or duration of follow-up.

**Table 2:** **Risk of colorectal cancer in relation to creatinine-adjusted urinary 1-OHPG concentration**^**a**^

**1-OHPG**^**b**^	**N**_**cases**_	**N**_**controls**_	**Unadjusted OR (95% CI)**	**Adjusted OR (95% CI) **^**c**^
Colorectal cancer				
Q1: <0.10	89	86	Ref	Ref
Q2: ≥0.10 to <0.196	100	86	1.11 (0.74-1.68)	1.11 (0.73-1.71)
Q3: ≥0.196 to <0.331	80	86	0.89 (0.58-1.37)	0.88 (0.56-1.38)
Q4: ≥0.331	74	85	0.84 (0.55-1.30)	0.85 (0.54-1.33)
			*P*_trend_ = 0.3	*P*_trend_ = 0.3
Per 1-unit increase in 1-OHPG^d^			0.98 (0.95-1.02)	0.98 (0.95-1.02)
Colon cancer				
Q1: <0.10	54	48	Ref	Ref
Q2: ≥0.10 to <0.196	59	53	0.96 (0.56-1.64)	0.93 (0.53-1.65)
Q3: ≥0.196 to <0.331	50	51	0.84 (0.47-1.50)	0.77 (0.41-1.46)
Q4: ≥0.331	42	53	0.70 (0.39-1.24)	0.69 (0.40-1.29)
			*P*_trend_ = 0.2	*P*_trend_ = 0.2
Per 1-unit increase in 1-OHPG^d^			0.98 (0.93-1.03)	0.98 (0.93-1.03)
Rectal cancer				
Q1: <0.10	35	38	Ref	Ref
Q2: ≥0.10 to <0.196	41	33	1.35 (0.70-2.61)	1.43 (0.70-2.93)
Q3: ≥0.196 to <0.331	30	35	0.95 (0.49-1.83)	0.93 (0.46-1.87)
Q4: ≥0.331	32	32	1.08 (0.56-2.09)	1.09 (0.54-2.21)
			*P*_trend_ = 0.9	P_trend_ = 0.9
Per 1-unit increase in 1-OHPG^d^			0.99 (0.92-1.07)	0.98 (0.91-1.07)

Abbreviations: *1-OHPG* 1-hydroxypyrene glucuronide, *OR* odds ratio, *CI* confidence interval.

^a^ Quartiles were determined based on the distribution of creatinine-adjusted 1-OHPG measurements among controls.

^b^ μmol/mol creatinine.

^c^ Adjusted for the following covariates: age, education, smoking status, aspirin use, fruit intake, vegetable intake, folate intake, measured body mass index (BMI), leisure time physical activity, and occupational physical activity.

^d^ A 1-unit increase was defined as half of the difference between the 25^th^ and 75^th^ percentiles for creatinine-adjusted 1-OHPG measurements among controls.

### Determinants of 1-OHPG levels

The geometric mean (95% CI) of creatinine-adjusted 1-OHPG concentrations by categories of selected exposures from the baseline questionnaire are reported in Table 3. Among 652 controls, the median (25^th^-75^th^ percentile) urinary 1-OHPG levels with and without adjustment for creatinine were 0.21 (0.11-0.38) μmol/mol creatinine and 2.01 (0.95-4.09) pmol/mL, respectively. Subjects who reported ever smoking had significantly higher 1-OHPG levels than non-smokers (*P* = 0.03). We observed a borderline significant association between husband’s smoking status and 1-OHPG concentration; women whose husbands were current smokers had higher levels of 1-OHPG than women whose husbands never smoked (*P* = 0.05). Results for passive smoking were similar when we restricted to women who never smoked. Mean 1-OHPG levels were approximately twice as high among subjects who reported using coal for cooking at their current residence compared to subjects who used gas or other types of fuel, though this difference did not achieve statistical significance (*P* = 0.08). Relative to subjects who ever ate stir-fried meats, those who did not had higher 1-OHPG levels; this difference was borderline significant (*P* = 0.05).

**Table 3:** Creatinine-adjusted urinary 1-OHPG concentration (μmol/mol creatinine) in relation to reported exposures from the baseline questionnaire

**Exposure**	**N**	**GM (95% CI)**	**P value**
**Overall**	652	0.18 (0.16-0.19)	
**Smoking variables**			
Ever smoked			
No	622	0.17 (0.16-0.19)	0.03
Yes	30	0.28 (0.18-0.44)	
Husband’s smoking status			
Never smoked	239	0.16 (0.14-0.19)	Ref
Former smoker	77	0.15 (0.11-0.21)	0.8
Current smoker	233	0.20 (0.17-0.23)	0.05
Not married	100	0.18 (0.14-0.23)	0.4
**Cooking variables**			
Type of cooking fuel used at current residence			
Gas or other besides coal	640	0.18 (0.16-0.19)	Ref
Coal	12	0.33 (0.17-0.64)	0.08
Cooking ventilation conditions			
Good	391	0.17 (0.16-0.20)	Ref
Fairly good	249	0.19 (0.16-0.22)	0.6
Poor	12	0.13 (0.05-0.33)	0.4
**Food consumption variables**			
Doneness of fried or baked meat or fish			
Not brown or never eat	104	0.18 (0.13-0.24)	Ref
Light brown	292	0.17 (0.15-0.20)	0.8
Dark brown	218	0.18 (0.16-0.21)	0.8
Entirely brown	11	0.27 (0.18-0.40)	0.4
Fried meats			
Never	207	0.19 (0.16-0.22)	0.5
Ever	445	0.17 (0.15-0.19)	
Stir-fried meats			
Never	20	0.30 (0.16-0.56)	0.05
Ever	632	0.17 (0.16-0.19)	
Roasted meats			
Never	206	0.20 (0.16-0.23)	0.2
Ever	446	0.17 (0.15-0.19)	
Smoked meats			
Never	439	0.18 (0.16-0.20)	0.8
Ever	213	0.17 (0.15-0.20)	

Abbreviations: *1-OHPG* 1-hydroxypyrene glucuronide, *GM* geometric mean, *CI* confidence interval.

We also evaluated creatinine-adjusted 1-OHPG concentration in relation to reported recent exposures from the sample collection questionnaire (Table 4). Similar to the results of the baseline questionnaire analysis, participant smoking was associated with elevated 1-OHPG levels. We observed statistically significant trends of increasing 1-OHPG concentration by number of cigarettes smoked in the last week (*P* = 0.03) and the last 24 hours (*P* = 0.04) prior to sample collection. Subjects who reported eating fried dough (yóutiáo) in the last 24 hours also had significantly higher 1-OHPG levels than subjects who did not eat these foods (*P* = 0.01). No other notable differences in 1-OHPG concentration in relation to recent exposures were observed.

**Table 4:** Creatinine-adjusted urinary 1-OHPG concentration (μmol/mol creatinine) in relation to recent exposures reported on the sample collection questionnaire

**Exposure**	**N**	**GM (95% CI)**	**P value**
**Smoking variables**			
In the last week			
No	626	0.17 (0.16-0.19)	0.10
Yes	26	0.26 (0.15-0.46)	
In the last 24 hours			
No	627	0.17 (0.16-0.19)	Ref
Yes	25	0.27 (0.15-0.48)	0.09
1-5 cigarettes	11	0.22 (0.09-0.55)	
6-10 cigarettes	9	0.22 (0.09-0.54)	P_trend_ = 0.04
>10 cigarettes	5	0.60 (0.05-6.73)	
Number of cigarettes in last week as a continuous variable	652	β = 0.008	0.03
Number of cigarettes in last 24 hours as a continuous variable	652	β = 0.053	0.04
**Food consumption variables**			
Fried dough products			
In the last week			
No	446	0.17 (0.15-0.19)	0.3
Yes	206	0.19 (0.16-0.23)	
In the last 24 hours			
No	587	0.17 (0.15-0.19)	0.01
Yes	65	0.26 (0.19-0.34)	
Fried meats			
In the last week			
No	500	0.18 (0.16-0.20)	0.9
Yes	152	0.18 (0.14-0.21)	
In the last 24 hours			
No	583	0.18 (0.16-0.20)	0.9
Yes	69	0.18 (0.14-0.24)	
Stir-fried or roasted meats			
In the last week			
No	110	0.19 (0.15-0.25)	0.4
Yes	542	0.17 (0.16-0.19)	
In the last 24 hours			
No	297	0.19 (0.16-0.22)	0.3
Yes	355	0.17 (0.15-0.19)	

Abbreviations: *1-OHPG* 1-hydroxypyrene glucuronide, *GM* geometric mean, *CI* confidence interval.

Results of multivariate analyses of creatinine-adjusted 1-OHPG concentration in relation to selected characteristics from the baseline questionnaire (Model 1) and reported exposures within the last 24 hours from the sample collection questionnaire (Model 2) are shown in Table 5. As in the bivariate analyses, participant smoking status was a statistically significant determinant of 1-OHPG concentration in both multivariate models. In Model 1, husband’s smoking status was associated with a slight, non-significant elevation in 1-OHPG concentration. Other factors in Model 1 that were associated with elevated levels of 1-OHPG included eating foods that were cooked well done (43% higher geometric mean relative to subjects who did not eat “browned” foods) and use of coal as a cooking fuel (63% higher geometric mean relative to subjects who used gas or other types of cooking fuel). In Model 2, recent consumption of fried dough products continued to be a significant determinant of 1-OHPG concentration after covariate adjustment (*P* = 0.01).

**Table 5:** Multivariate analyses of determinants of natural log-transformed creatinine-adjusted urinary 1-OHPG concentration

**Exposure**	**β**	**Percent difference in GM**	***P *value**
**Model 1: exposures from baseline questionnaire**^**b**^			
Smoking status			
Never	Ref		
Ever	0.501	65%	0.03
Husband’s smoking status			
No^c^	Ref		
Yes	0.148	16%	0.2
Doneness of fried or baked meat or fish			
Not brown or never eat	Ref		
Light brown	−0.016	−2%	0.9
Dark brown	0.002	0%	1.0
Entirely brown	0.357	43%	0.4
Type of cooking fuel used at current residence			
Gas or other besides coal	Ref		
Coal	0.491	63%	0.2
Cooking ventilation conditions			
Good	Ref		
Fairly good	0.014	1%	0.9
Poor	−0.334	−28%	0.4
**Model 2: exposures within the last 24 hours from sample collection questionnaire**^**b**^			
Number of cigarettes	0.062	6% per cigarette smoked	0.02
Ate fried dough products			
No	Ref		
Yes	0.406	50%	0.01
Ate fried meats			
No	Ref		
Yes	0.034	3%	0.8
Ate stir-fried or roasted meats			
No	Ref		
Yes	−0.087	−8%	0.4

Abbreviations: *1-OHPG* 1-hydroxypyrene glucuronide, *GM* geometric mean.

^a^ Estimated using the following formula: exp(β) - 1.

^b^ Adjusted for age, education, measured BMI, and study sample (colorectal or gastric cancer study).

^c^ Includes subjects who are not married, or whose husbands never smoked or formerly smoked.

Results of all analyses of colorectal cancer risk and determinants of 1-OHPG levels were similar when 1-OHPG concentration was not corrected for creatinine and when creatinine was included as a covariate in the statistical model (data not shown). We also performed analyses of colorectal cancer risk in relation to those exposures that were associated with elevated urinary 1-OHPG levels (e.g., personal smoking status, husband smoking status, consumption of fried dough products, and cooking with a coal stove), and no statistically significant associations were observed (not shown).

## Discussion

In this nested case–control study among participants in the SWHS, we did not find evidence of an association between urinary concentration of 1-OHPG, an established marker of PAH exposure, and risk of colorectal cancer. There is considerable evidence from both animal and human studies that PAHs and PAH-containing materials are carcinogenic [reviewed in ref. [19]. Potential exposure to PAHs from employment as a gas furnace worker [4], smoking [10], and reported meat consumption [5-9] has been linked to colorectal cancer and colorectal adenoma. Each of these risk factors might involve exposure to various pyrolysis compounds, though PAHs are suspected to be among the agents responsible for the association with colorectal cancer. In one case–control study, Sinha and colleagues [5] estimated dietary intake of benzo(a)pyrene (a PAH metabolite) and its relation with colorectal adenoma risk. The investigators reported an odds ratio of 5.6 comparing subjects in the highest quintile of estimated dietary benzo(a)pyrene intake relative to the lowest quintile, and a statistically significant dose–response trend was observed [5]. However, in a more recent large prospective study, estimated dietary intake of benzo(a)pyrene was not associated with colorectal cancer risk [20].

In the present study, we sought to further evaluate the hypothesis that PAH exposure is associated with an increased risk of colorectal cancer using urinary 1-OHPG concentration as a quantitative biological measure of PAH exposure. Very few studies have evaluated colorectal cancer risk in relation to biological markers of PAH exposure. A hospital-based case–control study of colorectal adenoma among non-smoking U.S. residents by Gunter et al. [11] characterized PAH exposure based on the level of leukocyte PAH-DNA adducts, which is considered to be a marker of relatively recent exposure i.e., within the last week; ref. [21]. In this case–control study, individuals in the highest quartile of leukocyte PAH-DNA adduct levels had a significantly greater risk of colorectal adenoma than individuals in the lowest quartile, and a statistically significant dose–response trend of increasing colorectal adenoma risk by quartile of PAH-DNA adduct level was observed. However, this study was limited by a relatively small number of cases of colorectal adenoma (N=82), and blood samples were collected after diagnosis. More recently, Agudo et al. [12] conducted a case-cohort study evaluating the relationship between aromatic DNA adduct levels in pre-diagnostic leukocytes and risk of colorectal cancer (154 cases). The investigators reported a statistically significant increased risk of colorectal cancer with increasing levels of aromatic DNA adducts, and observed a stronger association for colon cancer than for rectal cancer. In the present study, we were unable to confirm these findings using pre-diagnostic urinary 1-OHPG measurements in a larger sample of colorectal cancer cases and individually matched controls.

As a secondary analysis, we also evaluated potential determinants of urinary 1-OHPG concentration among 652 control subjects from the SWHS cohort. This sample was comprised of women ages 40 to 70 residing in Shanghai, China, only 4.6% of whom ever smoked cigarettes. In general, the levels of 1-OHPG in this population were considerably higher than what is typically observed among non-smokers in the United States, Europe and Korea, where median levels are approximately 0.3-0.4 pmol/mL [22,23]. The median (interquartile range) urinary concentration of 1-OHPG that we observed was 2.01 pmol/mL (0.95-4.09), which is comparable to levels observed in other populations in Linxian, China [24], northeastern Iran [25], and South Brazil [26], all of which are regions with well-characterized high levels of PAH exposure.

Results of bivariate and multivariate analyses revealed that active and passive smoking, cooking with coal as a fuel source, eating meat or fish that was cooked until it was “entirely brown”, and recent consumption of fried dough products influenced levels of 1-OHPG. However, only active smoking and recent consumption of fried dough products were associated with statistically significant increases in urinary 1-OHPG concentration after adjustment for other covariates. Active cigarette smoking has been consistently associated with higher urinary levels of 1-OHPG in various populations [22,25-27]. It is reasonable to expect that passive smoke exposure might also influence 1-OHPG levels. Our findings regarding passive smoking were consistent with the results of a previous study of determinants of urinary 1-OHPG concentration among children in South Korea, which found that the reported number of cigarettes smoked in the child’s home was associated with increased 1-OHPG concentration [28].

Diet, in particular consumption of charbroiled meats, has also been recognized as an important determinant of 1-OHPG concentration [1,23]. Lee et al. [28] reported that consumption of grilled fish, but not grilled meats, was associated with children’s’ levels of 1-OHPG. We found that the “doneness” of fried or baked meat or fish was associated with 1-OHPG concentration, though results were not statistically significant. Consumption of fried dough products – such as yóutiáo, a deep-fried twisted dough stick that is a popular breakfast food often purchased from street vendors or in snack shops – could potentially be an important dietary source of PAH exposure. High levels of benzo(a)pyrene have been detected in cooking oil fumes from shops selling yóutiáo [29], and it is possible that individuals might be exposed via ingestion of dough that has been fried in cooking oil containing PAHs. This finding suggests that consumption of fried dough foods such as yóutiáo should be considered in future studies that assess dietary PAH exposure in Chinese populations. Though very few subjects never ate stir-fried meats (N=20), we observed higher 1-OHPG levels in this group. This finding was unexpected, and no such association between 1-OHPG levels and recent consumption of stir-fried or roasted meats was observed. However, it is possible that this association may reflect other differences in diet, and further investigation of dietary PAH exposure among these individuals may reveal other potential sources of exposure.

### Strengths and limitations

The large sample size, prospective collection of urine samples, and availability of data on potential confounding factors were strengths of this study. Because samples were collected prospectively and results were similar when cases diagnosed within two years of sample collection were excluded, it is unlikely that early disease processes would have affected 1-OHPG measurements. The availability of prospectively collected data on covariates minimized potential recall bias and allowed us to control for potential confounding factors such as age, BMI, physical activity, and fruit and vegetable intake in our analyses.

There were also several limitations in this study. Urine was collected as a spot sample for practical reasons, though the interpretation of urinary creatinine levels used for the correction to ≥24 hour urine output may be problematic. To address this issue, we performed sensitivity analyses without correction for creatinine and with adjustment for creatinine concentration as a covariate in the statistical model; results were similar to what was observed in the main analyses.

As with most prospective investigations involving a single pre-diagnostic specimen, it is possible that urinary levels of 1-OHPG may not have reflected long-term PAH exposure status or exposure levels during the etiologically relevant period. In our analyses among controls, we identified associations between several reported sources of exposure from the baseline and sample collection questionnaires and urinary concentration of 1-OHPG, suggesting that 1-OHPG levels may be a reasonable reflection of both usual and recent exposure to PAHs. It should be noted that when we evaluated colorectal cancer risk in relation to these sources of PAH exposure, our findings were similarly null. The median time from urine sample collection until diagnosis of colorectal cancer in this study was 3.8 years; continued follow-up of the cohort may be useful to assess relations between long-term PAH exposure and colorectal cancer risk.

Most previous studies of determinants of 1-OHPG concentration have been based on relatively small sample sizes, and the inclusion of a large number of healthy subjects (N = 652) was a strength of the present study. However, despite the large sample size, some of the reported exposures were relatively uncommon in this population. Also, there was somewhat less heterogeneity of 1-OHPG levels relative to other highly exposed populations [25,26], which may have limited our ability to detect associations with colorectal cancer and to evaluate determinants of PAH exposure. We were unable to assess ambient exposure to PAHs in this study, an important determinant of exposure in previous studies [30] that could possibly explain the high and relatively homogeneous levels of 1-OHPG in our study population.

## Conclusions

We did not find evidence of an association between urinary 1-OHPG levels and colorectal cancer risk in this nested case–control study. Future studies with greater duration of follow-up and characterization of PAH exposure over a longer time period are needed to better understand the role of PAHs in the etiology of colorectal cancer.

## Abbreviations

1-OHPG: 1-hydroxypyrene glucuronide; BMI: Body mass index; CI: Confidence interval; CV: Coefficient of variation; GM: Geometric mean; MET-h/wk: Metabolic equivalent hours per week; OR: Odds ratio; PAH: Polycyclic aromatic hydrocarbon; QC: Quality control; SWHS: Shanghai Women’s Health Study.

## Competing interests

The authors declare that they have no competing financial interests.

## Authors’ contributions

JNH conducted the data analysis and drafted the manuscript; LML advised on the statistical analyses and helped draft the manuscript; PTS conducted the assays measuring urinary 1-OHPG levels and provided important intellectual content; XOS, GY, BTJ, HLL, NR, YTG, and WZ oversaw subject recruitment and data collection for the cohort study and provided intellectual input into preparation of the manuscript; FK and WHC conceptualized the study, advised on statistical analyses, and helped draft the manuscript. All authors read and approved the final manuscript.

## Pre-publication history

The pre-publication history for this paper can be accessed here:

http://www.biomedcentral.com/1471-2407/13/282/prepub

## References

[B1] Strickland P Kang D Sithisarankul P Polycyclic aromatic hydrocarbon metabolites in urine as biomarkers of exposure and effect Environ Health Perspect 1996 104 Suppl 5 927 932 10.1289/ehp.96104s5927 8933036 PMC1469694

[B2] IARC Polycyclic Aromatic Hydrocarbons IARC Monographs on the Evaluation of Carcinogenic Risks to Humans 2008 Lyon, France: International Agency for Research on Cancer

[B3] Parkin DM Bray FI Schottenfeld D, Fraumeni JFJr International Patterns of Cancer Incidence and Mortality Cancer Epidemiology and Prevention 2006 3 New York: Oxford University Press 101 138

[B4] Berger J Manz A Cancer of the stomach and the colon-rectum among workers in a coke gas plant Am J Ind Med 1992 22 825 834 10.1002/ajim.4700220605 1463028

[B5] Sinha R Kulldorff M Gunter MJ Strickland P Rothman N Dietary benzo[a]pyrene intake and risk of colorectal adenoma Cancer Epidemiol Biomarkers Prev 2005 14 2030 2034 10.1158/1055-9965.EPI-04-0854 16103456

[B6] Sinha R Peters U Cross AJ Meat, meat cooking methods and preservation, and risk for colorectal adenoma Cancer Res 2005 65 8034 8041 16140978 10.1158/0008-5472.CAN-04-3429

[B7] Gunter MJ Probst-Hensch NM Cortessis VK Meat intake, cooking-related mutagens and risk of colorectal adenoma in a sigmoidoscopy-based case–control study Carcinogenesis 2005 26 637 642 15579480 10.1093/carcin/bgh350

[B8] Fu Z Shrubsole MJ Smalley WE Association of meat intake and meat-derived mutagen exposure with the risk of colorectal polyps by histologic type Cancer Prev Res (Phila) 2011 4 1686 1697 10.1158/1940-6207.CAPR-11-0191 21803984 PMC3188364

[B9] Ferrucci LM Sinha R Huang WY Meat consumption and the risk of incident distal colon and rectal adenoma Br J Cancer 2012 106 608 616 10.1038/bjc.2011.549 22166801 PMC3281548

[B10] Botteri E Iodice S Bagnardi V Smoking and colorectal cancer: a meta-analysis JAMA 2008 300 2765 2778 10.1001/jama.2008.839 19088354

[B11] Gunter MJ Divi RL Kulldorff M Leukocyte polycyclic aromatic hydrocarbon-DNA adduct formation and colorectal adenoma Carcinogenesis 2007 28 1426 1429 10.1093/carcin/bgm022 17277232

[B12] Agudo A Peluso M Munnia A Aromatic DNA adducts and risk of gastrointestinal cancers: a case-cohort study within the EPIC-Spain Cancer Epidemiol Biomarkers Prev 2012 21 685 692 10.1158/1055-9965.EPI-11-1205 22315368

[B13] Strickland PT Kang D Bowman ED Identification of 1-hydroxypyrene glucuronide as a major pyrene metabolite in human urine by synchronous fluorescence spectroscopy and gas chromatography–mass spectrometry Carcinogenesis 1994 15 483 487 10.1093/carcin/15.3.483 8118933

[B14] Zheng W Chow WH Yang G The Shanghai Women's Health Study: rationale, study design, and baseline characteristics Am J Epidemiol 2005 162 1123 1131 10.1093/aje/kwi322 16236996

[B15] Kamangar F Diaw L Wei WQ Serum pepsinogens and risk of esophageal squamous dysplasia Int J Cancer 2009 124 456 460 10.1002/ijc.23918 18844222 PMC2605159

[B16] Matthews CE Shu XO Jin F Lifetime physical activity and breast cancer risk in the Shanghai Breast Cancer Study Br J Cancer 2001 84 994 1001 10.1054/bjoc.2000.1671 11286483 PMC2363839

[B17] Han IK Duan X Zhang L 1-Hydroxypyrene concentrations in first morning voids and 24-h composite urine: intra- and inter-individual comparisons J Expo Sci Environ Epidemiol 2008 18 477 485 10.1038/sj.jes.7500639 18059422

[B18] Barr DB Wilder LC Caudill SP Urinary creatinine concentrations in the U.S. population: implications for urinary biologic monitoring measurements Environ Health Perspect 2005 113 192 200 15687057 10.1289/ehp.7337 PMC1277864

[B19] Bostrom CE Gerde P Hanberg A Cancer risk assessment, indicators, and guidelines for polycyclic aromatic hydrocarbons in the ambient air Environ Health Perspect 2002 110 Suppl 3 451 488 12060843 10.1289/ehp.110-1241197 PMC1241197

[B20] Cross AJ Ferrucci LM Risch A A large prospective study of meat consumption and colorectal cancer risk: an investigation of potential mechanisms underlying this association Cancer Res 2010 70 2406 2414 10.1158/0008-5472.CAN-09-3929 20215514 PMC2840051

[B21] Rothman N Correa-Villasenor A Ford DP Contribution of occupation and diet to white blood cell polycyclic aromatic hydrocarbon-DNA adducts in wildland firefighters Cancer Epidemiol Biomarkers Prev 1993 2 341 347 8348057

[B22] Kang D Rothman N Cho SH Association of exposure to polycyclic aromatic hydrocarbons (estimated from job category) with concentration of 1-hydroxypyrene glucuronide in urine from workers at a steel plant Occup Environ Med 1995 52 593 599 10.1136/oem.52.9.593 7550799 PMC1128312

[B23] Kang DH Rothman N Poirier MC Interindividual differences in the concentration of 1-hydroxypyrene-glucuronide in urine and polycyclic aromatic hydrocarbon-DNA adducts in peripheral white blood cells after charbroiled beef consumption Carcinogenesis 1995 16 1079 1085 10.1093/carcin/16.5.1079 7767968

[B24] Roth MJ Qiao YL Rothman N High urine 1-hydroxypyrene glucuronide concentrations in Linxian, China, an area of high risk for squamous oesophageal cancer Biomarkers 2001 6 381 386 10.1080/13547500110044780 23889311

[B25] Kamangar F Strickland PT Pourshams A High exposure to polycyclic aromatic hydrocarbons may contribute to high risk of esophageal cancer in northeastern Iran Anticancer Res 2005 25 425 428 15816606

[B26] Fagundes RB Abnet CC Strickland PT Higher urine 1-hydroxy pyrene glucuronide (1-OHPG) is associated with tobacco smoke exposure and drinking mate in healthy subjects from Rio Grande do Sul, Brazil BMC Cancer 2006 6 139 10.1186/1471-2407-6-139 16729889 PMC1539013

[B27] Hu Y Zhou Z Xue X Sensitive biomarker of polycyclic aromatic hydrocarbons (PAHs): urinary 1-hydroxyprene glucuronide in relation to smoking and low ambient levels of exposure Biomarkers 2006 11 306 318 10.1080/13547500600626883 16908438

[B28] Lee KH Vermeulen R Lenters V Cho SH Strickland PT Kang D Determinants of urinary 1-hydroxypyrene glucuronide in South Korean children Int Arch Occup Environ Health 2009 82 961 968 10.1007/s00420-008-0385-2 19020893

[B29] Li S Pan D Wang G Analysis of polycyclic aromatic hydrocarbons in cooking oil fumes Arch Environ Health 1994 49 119 122 10.1080/00039896.1994.9937464 8161241

[B30] Huang HB Lai CH Chen GW Traffic-related air pollution and DNA damage: a longitudinal study in Taiwanese traffic conductors PLoS One 2012 7 5 e37412 10.1371/journal.pone.0037412 22629390 PMC3357412

